# Seromyotomy as an alternative to gastric bypass for post-sleeve gastrectomy stricture: a case report

**DOI:** 10.1093/jscr/rjag296

**Published:** 2026-04-23

**Authors:** Kevin Connors, Carolina Baz, Alejandro Gandsas

**Affiliations:** Department of Surgery, Luminis Health Anne Arundel Medical Center, 2001 Medical Parkway, Annapolis, MD 21401, United States; Department of Surgery, Luminis Health Anne Arundel Medical Center, 2001 Medical Parkway, Annapolis, MD 21401, United States; Department of Surgery, Luminis Health Anne Arundel Medical Center, 2001 Medical Parkway, Annapolis, MD 21401, United States

**Keywords:** sleeve gastrectomy, gastric stricture, gastric stenosis, seromyotomy

## Abstract

Laparoscopic sleeve gastrectomy (LSG) is widely performed, but postoperative strictures, most commonly at the incisura angularis, remain a challenging complication. These are often functional, resulting from angulation of the gastric tube. While endoscopic balloon dilation is the first-line therapy, some patients require surgical intervention. Conversion to Roux-en-Y gastric bypass (RYGB) is traditionally the default treatment. Laparoscopic seromyotomy offers an anatomy-preserving alternative in select cases. We report a 35-year-old female with prior LSG and paraesophageal hernia repair who developed progressive food intolerance due to a functional incisura stricture. Endoscopy demonstrated sharp luminal angulation without fixed narrowing. Given the patient’s preference to avoid RYGB, a diagnostic laparoscopy with seromyotomy was performed. Postoperative imaging confirmed resolution of the stricture without leak. The patient tolerated a solid diet and remained asymptomatic without reflux at 2-year follow-up. Laparoscopic seromyotomy is a viable option for carefully selected patients with refractory functional strictures following LSG.

## Introduction

Laparoscopic sleeve gastrectomy (LSG) has become one of the most performed bariatric procedures due to its effectiveness in weight loss and metabolic improvements with long term durability [1, 2]. However, postoperative complications such as strictures remain a significant concern, with reported incidences ranging from 0.69% to 4% [3–5]. Strictures typically occur at the incisura angularis due to oversewing the staple line, twisting, or excessive tissue resection [4, 6]. While endoscopic balloon dilation (EBD) is considered the first-line treatment, up to 44% of patients experience residual symptoms requiring either repeat dilation or surgical intervention [6]. Traditionally, conversion to Roux-en-Y gastric bypass (RYGB) has been the most popular approach for intractable stenosis [7]. While uncommon, alternative techniques such as laparoscopic seromyotomy can treat strictures without the complexities or complications of RYGB [8]. We present a patient with a symptomatic post-LSG stricture successfully treated with laparoscopic seromyotomy, highlighting its role as an effective surgical option for patients.

## Case presentation

This case describes a 35-year-old female who underwent LSG with concomitant paraesophageal hernia (PEH) repair 6 months prior and presented with progressive food intolerance. Her PEH and LSG were both performed over a 40 French bougie. She was able to tolerate consistencies up to puree but experienced nausea and emesis with anything thicker. These symptoms were concerning for a gastric stricture, and she underwent an esophagogastroduodenoscopy (EGD) 5 months postoperatively. The EGD demonstrated an acute angle of the gastric tube lumen at the level of the incisura angularis consistent with a functional stricture. After further discussion with the patient, she was hesitant to undergo conversion to RYGB and was agreeable to a diagnostic laparoscopy with possible seromyotomy versus conversion to RYGB to treat her symptoms and functional stricture.

**Figure 1:**
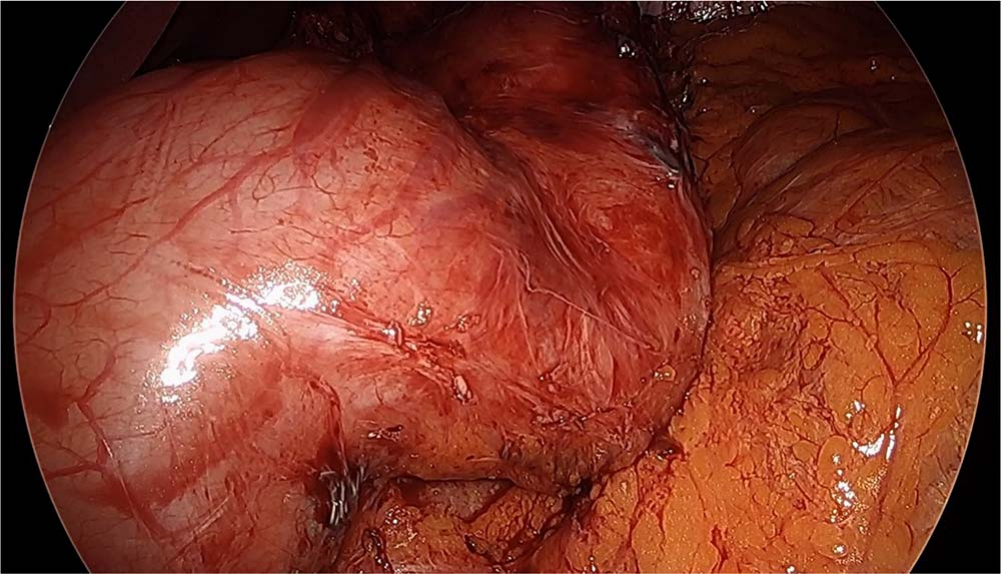
Stricture of gastric sleeve after lysis of adhesions.

Upon initial laparoscopy, the patient had significant adhesions between the stomach, liver, and omentum. These adhesions to the liver and omentum were taken down with a combination of ultrasonic shears, sharp, and blunt dissection to further mobilize the stomach. An endoscope was passed, demonstrating the area of functional stricture, which was also seen via laparoscopy (Fig. 1). The patient also had a recurrent PEH.

The dissection was carried out along the entire left side of the stomach to the esophagus. The stomach and esophagus were dissected off the left crus, and the dissection was extended anteriorly to the right crus for full mobilization of the proximal stomach. The dissection was taken up into the thorax to increase the intra-abdominal esophageal length. The stomach and esophagus had no tension in the abdomen, with ~4 cm of intra-abdominal esophageal length. The crura were closed with two additional interrupted 2-0 Ethibond sutures over a 40 French bougie.

**Figure 2:**
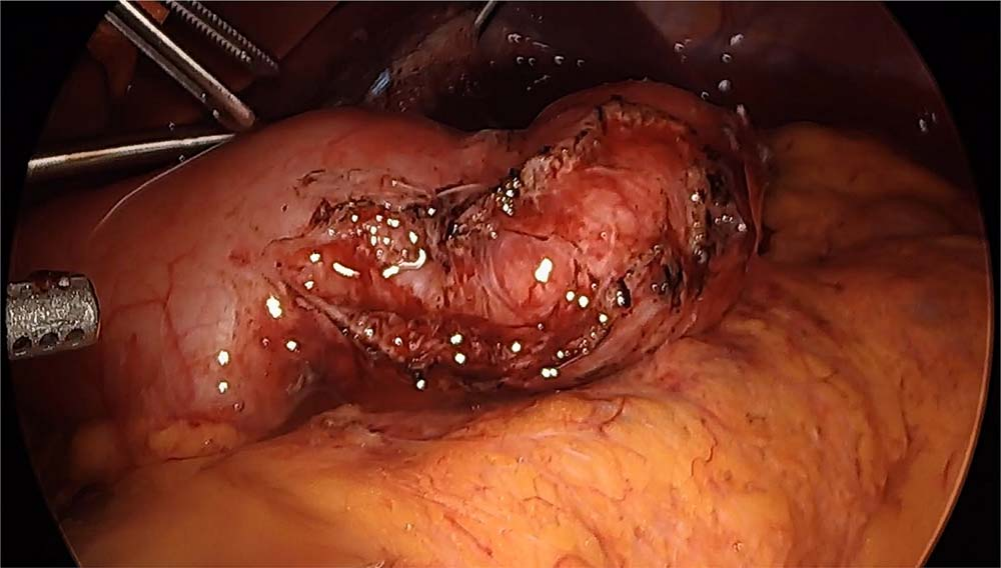
Gastric sleeve after seromyotomy and resolution of stricture.

The seromyotomy was then performed at the incisura angularis. The serosa was incised with hook cautery, and the longitudinal and circular muscularis fibers were divided with ultrasonic shears. The dissection was extended 3 cm proximally and distally from the strictured segment until mucosal bulging occurred, being careful not to violate the mucosa (Fig. 2).

The endoscope was passed easily with resolution of the previous stricture. The stomach was insufflated under irrigation, confirming a negative leak test. Hemostasis was achieved and a fibrin glue sealant was sprayed over the operative field.

**Figure 3:**
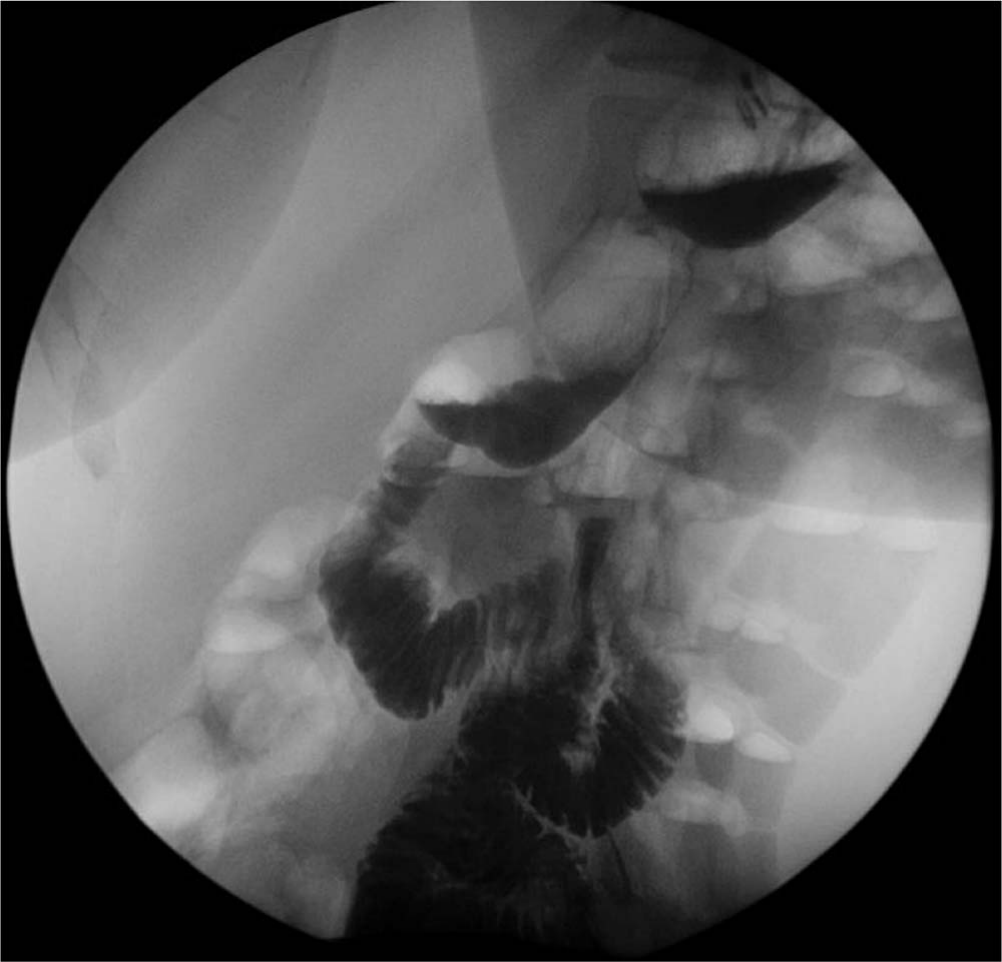
Upper GI study demonstrating resolution of stricture.

Postoperatively, a fluoroscopic upper gastrointestinal series was performed with no stricture identified and contrast passing easily through (Fig. 3). She continued to do well, tolerating a solid diet without significant reflux at the 2-year follow-up.

## Discussion

The literature supports a tiered approach to stricture management, beginning with minimally invasive interventions before progressing to more extensive surgical revisions. Endoscopic techniques, including EBD and stenting, have shown high initial success rates, with EBD providing symptomatic relief in 78%–96% of cases [9, 10]. However, recurrence remains a concern, particularly for functional strictures where a sharp angulation of the gastric tube is the primary issue [10]. Stenting offers a temporary solution for persistent cases, but complications such as migration and tissue overgrowth often limit its long-term effectiveness [11].

Surgical alternatives, such as laparoscopic seromyotomy, offer a promising option for select patients with gastric strictures who seek to preserve their gastric anatomy while effectively relieving obstruction. This technique involves selective division of the seromuscular layers of the gastric wall at the site of stenosis, thereby reducing luminal resistance while preserving the integrity of the mucosa [8]. It is critical when performing a seromyotomyto divide through both the longitudinal and circular muscle fibers, visualizing the bulging mucosa. This division must be taken proximal and distal to the point of stricture. If the mucosa is violated, conversion to a stricturoplasty or RYGB may be performed, and this should be included in the consent process. Dapri *et al.* demonstrated a reduction in dysphagia in a series of nine patients with only one gastric leak managed by stenting [8]. Vilallonga *et al.* reported a high leak rate (35%) in 14 patients following seromyotomy, highlighting the associated risks and emphasizing the need for advanced technical skill and a low threshold for mucosal injury concerns [5]. In our case, laparoscopic seromyotomy successfully resolved the patient’s symptomatic stricture. This resulted in the resolution of the stricture and allowed long-term tolerance of solid foods without reflux at the 2-year follow-up.

This case contributes to the literature supporting laparoscopic seromyotomy as a viable alternative for select patients with refractory post-LSG strictures. The patient’s long-term symptomatic resolution without the complexities of RYGB highlights the advantages of this approach. Further studies with larger cohorts are needed to better define patient selection criteria.
